# The Influence of the Size of BN NSs on Silkworm Development and Tissue Microstructure

**DOI:** 10.3390/nano13091502

**Published:** 2023-04-27

**Authors:** Vivian Andoh, Haiyan Liu, Liang Chen, Lin Ma, Keping Chen

**Affiliations:** 1 School of Food and Biological Engineering, Jiangsu University, Zhenjiang 212013, China; vandoh@ujs.edu.cn; 2 School of Life Sciences, Jiangsu University, Zhenjiang 212013, China; oochen@ujs.edu.cn; 3 College of Tea and Food Science Technology, Jiangsu Vocational College of Agriculture and Forestry, Jurong 212400, China; yanliuhai@126.com; 4 College of Biotechnology, Jiangsu University of Science and Technology, Zhenjiang 212000, China

**Keywords:** boron nitride nanosheets, size effect, silkworm development, tissue microstructure

## Abstract

Boron nitride nanosheets (BN NSs) have emerged as promising materials in a wide range of biomedical applications. Despite the extensive studies on these bio-nano interfacial systems, one critical concern is their toxicity, which is affected by a variety of factors, including size. This study aimed at assessing the relationship between BN NSs size and toxicity. Two silkworm strains (qiufeng × baiyu and Nistari 7019) were used as model organisms to investigate the effect of different sizes of BN NSs (BN NSs-1, thickness of 41.5 nm and diameter of 270.7 nm; BN NSs-2, thickness of 48.2 nm and diameter of 562.2 nm) on silkworm mortality, growth, cocoon weight, and tissue microstructure. The findings show that exposure to BN NSs in this work has no lethal adverse effects on silkworm growth or tissue microstructure. BN NSs have a higher effect on the growth rate of qiufeng × baiyu compared to Nistari 7019, demonstrating that the same treatment does not favorably affect the Nistari 7019 strain, as there is no significant increase in cocoon weight. Overall, the study suggests that the sizes of BN NSs employed in this study are relatively safe and have less negative impact on silkworms. This offers significant insights into the effect of BN NSs size, a crucial factor to consider for their safe use in biomedical applications.

## 1. Introduction

Boron nitride nanosheets (BN NSs) are two-dimensional nanomaterials with the same structure and properties as graphene but with a white hue; thus, they are also referred to as “white graphene” [1,2]. While BN NSs and graphene have many qualities, including a high surface area, superior thermal conductivity, and high mechanical strength, BN NSs have the advantage of insulating; thus, they can be employed in applications where electrical conductivity is not desirable [3,4]. In recent years, BN NSs have gained particular interest due to their good biocompatibility and excellent chemical, mechanical and thermal properties [5]. BN materials are already used in insulating substrates, multifunctional composite materials, optoelectronic nanodevices [6,7,8,9], etc. These also have lower cytotoxicity because of their chemical inertia, which explains their great potential in biomedical applications [10], despite their structural regulation and functional designs necessitating several toxic and corrosive chemicals. Even though there are several proposed applications of BN NSs and their derivatives, particularly their potential use in biomedical applications [11], there are limited reports on their biocompatibility and toxicity, especially their potential toxicity to humans and the environment, which is rarely discussed, and significantly underestimated.

While there have been some studies on the toxicity of BN NSs in living cells, the results have been inconsistent. Many challenges remain to be addressed, particularly those associated with their toxicity. Pan et al. [12] investigated the toxicity of BN NSs in mammalian cell lines. Their findings demonstrated that the mammalian cells could adapt to concentrations as high as 500 µg/mL. They also found that BN NSs caused minimal ROS generation and minor cytokine release. It was also revealed that BN NSs could be a promising drug delivery system for cancer treatment as they protect against free Camptothecin (CPT) toxicity and enhance their anti-cancer efficacy [13]. Yet, another study showed that hexagonal boron nitride nanosheets (hBN NSs) functionalized with hydroxyl groups were non-cytotoxic to L929 mouse cells, insect hemocytes, and human erythrocytes [14], suggesting poor reactivity with biological systems.

It is critical to understand that the toxicity of BN NSs can be influenced or regulated by several factors, including their sizes, shape, surface charge, and chemical composition, as well as the route of exposure, time, and dose [15,16,17]. A number of studies have reported the influence of BN NSs sizes on their effect. For example, a study demonstrated that cancerous MCF-7 and HeLa cells were more susceptible to the cytotoxic effects of nanostructured hBN of size 75 nm administered at a concentration of 2 mg/mL [18] and that with an average size of 472.7 nm, decreased cell viability caused detrimental effects on intracellular ROS production, mitochondrial depolarization and membrane integrity of human hepatoma HepG2 cells [19]. Meanwhile, polyethylene glycol boron nitrides (PEG-BNs) administered intravenously to mice caused tissue lesions [20]. In our previous study, BN NSs with an irregular sheet-like shape with an average diameter of 1.8 µm at concentrations ranging from 1–4% fed to silkworms had no adverse effect on their growth and tissues; however, significant changes in the expression of genes in the midgut involved in some specific functions were observed, indicating a potential risk of BN NSs causing dysfunction [21]. This could have implications for the long-term health and survival of the silkworms, as well as the safety of using BN NSs in other organisms or human applications. More research is needed to understand the potential risks associated with using BN materials to fully understand the mechanisms and to determine the potential risks related to their use. Additionally, the effect of long-term exposure to these materials still needs to be investigated. Nonetheless, the current literature on the toxicity and size effect of BN NSs is limited. Therefore, it is crucial to carefully consider these factors when conducting toxicity studies and interpreting the results.

Although BN NSs have excellent properties, such as their biocompatibility, compared to their synthesis, only a few studies have tackled their application on animal models such as insects. Silkworm (*Bombyx mori*) is an insect belonging to the Bombycoidea family within the Lepidoptera order. The primary purpose of silkworm-rearing was silk production; however, they have emerged as an important experimental model for various research fields. One of the advantages of using silkworms as an experimental model is that there are no ethical issues associated with their use, unlike with other animal models. Additionally, silkworms are easy to handle and manipulate. They can be reared in large quantities using established methods of feeding and maintenance and are relatively inexpensive compared to other animal models. Silkworms are also sensitive to toxicants, which makes them a valuable tool for environmental monitoring and toxicity studies [22,23,24,25]. They have been used to study the toxic effects of various chemicals and pollutants. Their responses to these substances can provide insights into the potential environmental and human health risks.

This study evaluated the effect of BN NSs size using two silkworm strains (qiufeng × baiyu, Nistari 7019) as model organisms by characterizing their growth status and tissue morphology. The length and weight of the larvae and cocoon and microstructures of several tissues represented the key parameters assessed. In general, the study indicates that the BN NSs utilized in this research are reasonably harmless and have a minor adverse effect on silkworms. This provides valuable information regarding the importance of the size of BN NSs, which is a critical consideration for their safe application in biomedical fields.

## 2. Materials and Methods

### 2.1. Materials and Reagents

Boron nitride nanosheets (BN NSs) were purchased from Beijing Deke Daojin Science and Technology Co., Ltd. (Beijing, China). *Bombyx mori* silkworm eggs from two strains (qiufeng × baiyu and Nistari 7019) were sourced from the College of Biotechnology from Jiangsu University of Science and Technology, Jiangsu, China.

### 2.2. Chemical Characterization

The morphology, diameter, thickness, energy-dispersive X-ray spectra (EDS), and size distribution of BN NSs were characterized by Shanghai Yanxi Analysis and Testing Technology Co. Ltd. (Shanghai, China). A scanning electron microscopy (SEM, Zeiss Merlin Compact, Jena, Germany) was used to study the morphology, diameter, thickness, and EDS of BN NSs; a Zetasizer Nano ZS ZEN3600 particle size analyzer (Malvern, UK) was used to perform the dynamic light scattering (DLS) to study the size distribution of BN NSs. A JEOL-2010 electron microscope from JEOL Ltd. recorded transmission electron microscopy (TEM, Tokyo, Japan) characterization.

### 2.3. Preparation of BN NSs Solution

Two different sizes of BN NSs were used, designated as BN NSs-1 (thickness of 41.5 nm, average diameter of approximately 200 nm) and BN NSs-2 (thickness of 48.2 nm, average diameter of approximately 500 nm), respectively. The BN NSs-1 (4 g) and BN NSs-2 (4 g) were mixed with 100 mL distilled water to form a cloudy suspension, then ultrasonicated for 30 min to ensure the solution was evenly dispersed. For the concentration and time investigation, 2% (2 g) and 8% (8 g) of BN NSs-1 and BN NSs-2 were used. These concentrations were used to establish a baseline understanding of how low concentrations of the two BN NSs affect the different strains of the silkworm. In addition, beginning with a small concentration range could ensure the detection of any significant differences in growth, development, and other parameters, which can inform future research involving a wider concentration range.

### 2.4. The Intake of BN NSs by Silkworms and Cocooning

Silkworm eggs were incubated in a conducive environment under a temperature of 25 °C and a relative humidity of approximately 75% until they hatched. Afterward, the silkworm larvae from the 1st to the 4th instar were fed fresh mulberry leaves only. On the 1st day of the 5th instar, 40 healthy and uniform-sized silkworm larvae of the strain qiufeng × baiyu strain were randomly chosen, divided evenly into two groups and fed with modified diets of fresh mulberry leaves sprayed with BN NSs solutions, respectively, until cocooning. The two groups were designated G1 and G2 according to the type of BN NSs they took in: (BN NSs-1: -4%; BN NSs-2: -4%). The larvae were fed twice daily, once in the morning and at dusk. Another 20 silkworms (qiufeng × baiyu) fed with mulberry leaves only were set as control: 0%. The same experimental procedure was carried out on the silkworm strain Nistari 7019. The weight of each larva was recorded from the 1st day of the 5th instar. After cocooning, the cocoons obtained from each group were then weighed. The silkworms were fed twice daily. The first four instars of silkworm larvae correspond to the stages of growth and development, during which the larvae undergo molting and increase in size. However, the 5th instar is a critical stage in the silkworm’s life cycle, as it marks the beginning of the intense feeding phase, which is necessary for silk production. By selecting healthy and uniformly sized 5th instar silkworm larvae, it is ensured that the experimental groups are comparable and that any observed differences in the growth and cocoon production of the silkworm will be attributed to the modified diets.

### 2.5. Histopathological Evaluation of Silkworm Tissues

On the 6th day of exposure to BN NSs, just before cocooning began, four larvae from each group were randomly selected and dissected [26] for pathological tests. Different tissues of silkworm larvae (midgut, fat body, and posterior silk gland) were removed and stored in 4% formalin. They were then cut into histological sections and mounted onto glass slides at Zhenjiang First People’s Hospital. Following hematoxylin-staining, a microscope (Leica EZ4HD, Leica Microsystems GmbH, Germany) was used to observe the cell morphology. 

### 2.6. Statistical Analysis

A one-way analysis of variance was used, followed by an unpaired two-tailed Student’s *t*-test program to perform statistical analyses. A *p*-value of less than 0.05 (*p* < 0.05) was considered statistically significant.

## 3. Results and Discussions

### 3.1. Characteristics of the Two Distinct Sizes of BN NSs

**Figure 1:**
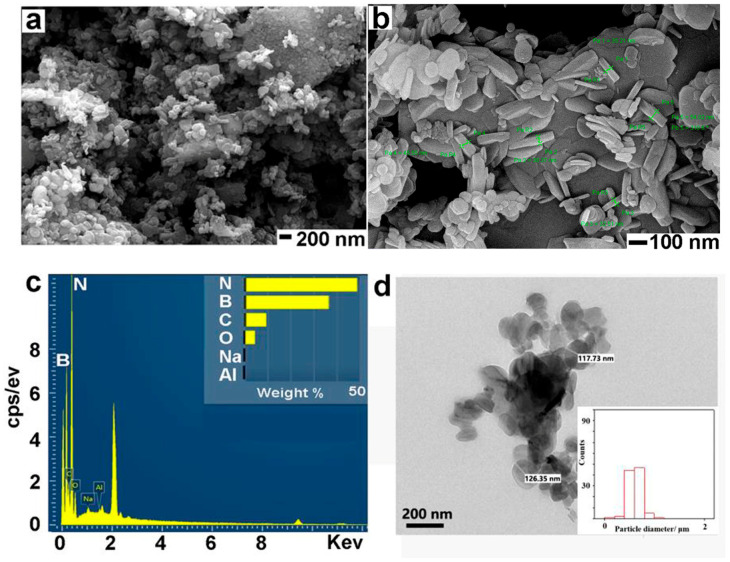
SEM images of BN NSs-1, captured in different directions and magnifications (**a**) and (**b**). EDS of the BN NSs-1 (**c**) and TEM of BN NSs-1 (**d**).

**Figure 2:**
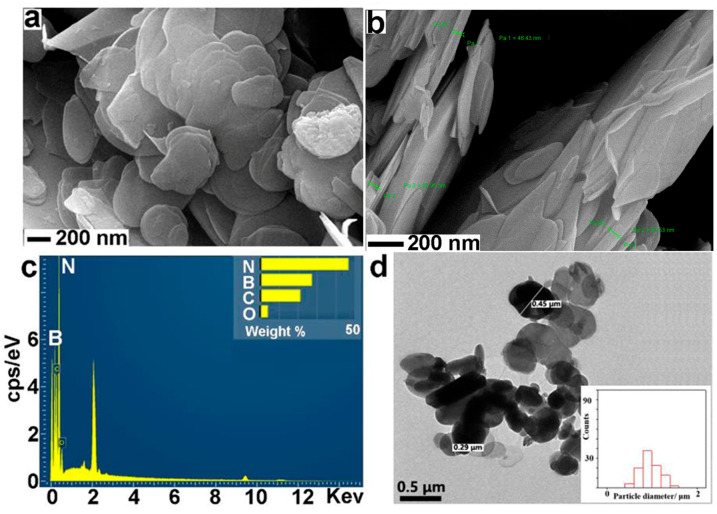
SEM images of BN NSs-2, captured in different directions and magnifications (**a**) and (**b**). EDS of BN NSs-2 (**c**) and TEM of BN NSs-2 (**d**).

The SEM images show that BN NSs-1 (Figure 1a,b) and BN NSs-2 (Figure 2a,b) both have sheet-like structures, indicating the existence of BN NSs. The SEM-EDS results show that the materials are primarily composed of B (boron) and N (nitrogen) elements (Figure 1c and Figure 2c). The thickness of BN NSs-1 was approximately 41.5 nm, while that of BN NSs-2 was approximately 48.2 nm. The diameter of BN NSs-1 was approximately 200 nm, while that of BN NSs-2 was approximately 0.5 μm (Figure 1d and Figure 2d). Dynamic light scattering (DLS) was carried out to determine the hydrodynamic size of the BN NSs. The diameter of BN NSs-1 ranged from 164.2 nm to 458.7 nm, with an average diameter of 270.7 nm. Similarly, BN NSs-2 ranged from 458.7 nm to 712.4 nm, with an average diameter of 562.2 nm. The solution for DLS was distilled water. BN NSs-1 and BN NSs-2 have polydispersity indices (PDI) of 0.295 and 0.863, respectively. Detailed information on the DLS can be found in Figure S1 in the Supplementary Materials.

### 3.2. The Effect of BN NSs on Larvae Growth and Mortality of Silkworm

**Figure 3:**
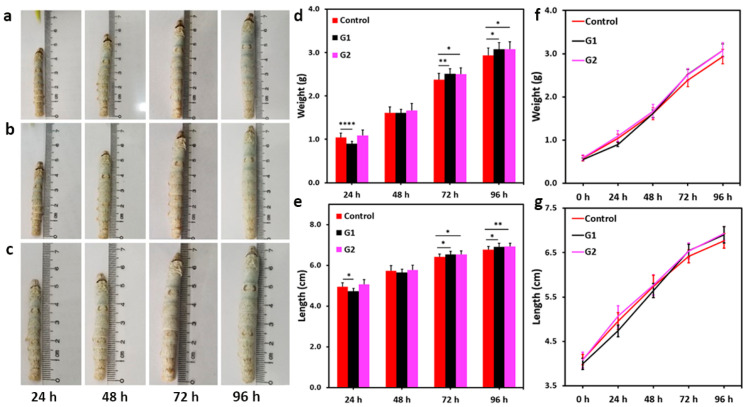
The effect of BN NSs on the growth of qiufeng × baiyu silkworms. (**a**–**c**) The images of silkworm larvae after the intake of BN NSs for different times; (**a**): control, (**b**): G1, (**c**): G2). (**d**,**e**) The comparison of the average weight (**d**) and length (**e**) of silkworm larvae from different groups; the error bars stand for the standard deviation of average weight and average length, respectively. (**f**,**g**) the growth trend of silkworm weight (**f**) and length (**g**) from different groups. Error bars presented in mean ± SD; * *p* < 0.05, ** *p* < 0.01, and **** *p* < 0.0001.

**Figure 4:**
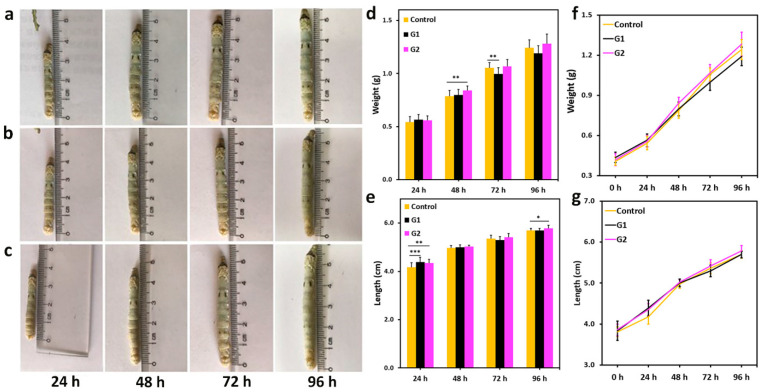
The effect of BN NSs on the growth of Nistari 7019 silkworms. (**a**–**c**) The images of silkworm. (**c**): G2). (**d**,**e**) The comparison of the average weight (**d**) and length (**e**) of silkworm larvae from different groups. The error bars represent the average weight and length standard deviation, respectively. (**f**,**g**) The growth trend of silkworm weight (**f**) and length (**g**) from different groups. Error bars presented in mean ± SD; * *p* < 0.05, ** *p* < 0.01, and *** *p* < 0.001.

Sixty silkworms each of the qiufeng × baiyu and Nistari 7019 strains were evenly divided into three groups, control, G1, and G2, respectively, based on their diet components (control: only mulberry leaves; G1: mulberry leaves sprayed with BN NSs-1 (4%); G2: mulberry leaves sprayed with BN NSs-2 (4%)). The silkworms’ physical appearance, weight, and length were recorded daily from the onset of BN NSs intake until the cocooning stage. The growth trend of the larvae of qiufeng × baiyu (Figure 3) and Nistari 7019 (Figure 4) was compared to that of the control group. It was observed that the larvae from all the groups (Figure 3a–c and Figure 4a–c) had a uniform and smooth longitudinal appearance. The average weight of qiufeng × baiyu showed positive growth (Figure 3d) as the days progressed; however, the average length (Figure 3e) was comparable across all groups. Meanwhile, the average weight of Nistari 7019 larvae G1 was lower than that of the control and G2 at 72 and 96 h (Figure 4d). Similar growth patterns in both silkworm strains are presented in Figure 3f,g and Figure 4f,g. It is well-known that the nanomaterial’s size could significantly impact the determination of their effects on biological systems. Smaller BN NSs (<50 nm) have been shown to have higher toxicity than larger ones, likely due to their increased surface area and higher reactivity [15]. Despite these observations, there were no mortality records, suggesting that both BN NSs had no significant negative impact on silkworm development and survival.

To further analyze and verify whether the data obey normal distribution, such as the Skewness Kurtosis test, SPSS software was used. Previous studies have shown that when the confidence level is 0.05 (α = 0.05), the skewness value falls within the range between -1.095 and + 1.095, and the Kurtosis value falls within the range between −2.191 and +2.191; it can be considered an approximately normal distribution [27]. Figures S2–S5 showed that most of the data from both strains of silkworm larvae (qiufeng baiyu and Nistari 7019), including silkworm weight and length, followed the normal distribution. However, a few data points did not follow the normal distribution, including the weight and length of qiufeng baiyu silkworm larvae at 0 h (control), the weight of Nistari 7019 silkworm larvae at 48 h (control), and the length of Nistari 7019 silkworm larvae at 48 h (control). 

The nonparametric test, such as the Kruskal–Wallis Test [28,29], was then used to verify whether there were significant differences among the three groups of silkworms treated with the two different-sized BN NSs and the control group. From the data of qiufeng × baiyu (Table S2), there were no significant differences among the three groups of silkworm weight at 0 h; however, significant differences were observed at 24, 72, and 96 h. Similarly, the same phenomenon was observed for the silkworm length of qiufeng × baiyu (Table S5). For Nistari 7019 (Tables S4 and S6), there were no significant differences among the three groups of silkworm weight at 0 and 24 h, but significant differences were observed at 48, 72, and 96 h. The same phenomenon was observed for the silkworm length of Nistari 7019. The results suggest substantial differences between the two groups compared to the control, likely due to the two silkworm strains’ different biological characteristics and sensitivities to various nanomaterials [30,31]. However, there was no record of deaths during the experiment. The food intake speed and growth were similar across all groups, indicating that the two distinct sizes of BN NSs had no or less negative effect on the silkworm. The raw data on the food intake speed of silkworms from different groups are shown in Table S7 in the Supplementary Materials.

**Figure 5:**
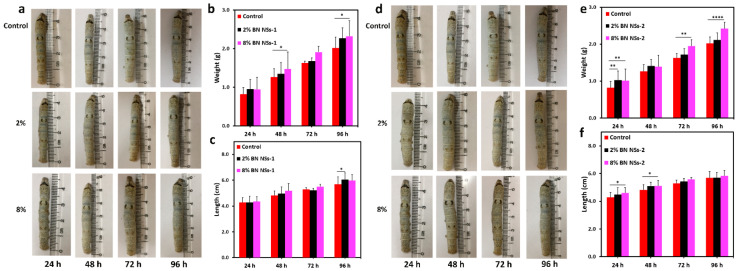
The effect of BN NSs-1 and 2 on the growth of silkworms (qiufeng × baiyu) at different concentrations. (**a**) The images of silkworm larvae after the intake of BN NSs-1. (**b**,**c**) The comparison of the average weight (**b**) and average length (**c**) from different groups. (**d**) The images of silkworm larvae after the intake of BN NSs-2. (**b**,**c**) The comparison of the average weight (**e**) and average length (**f**) from different groups. Error bars presented in mean ± SD; * *p* < 0.05, ** *p* < 0.01, and **** *p* < 0.0001.

This study further investigated the concentration and time effect of BN NSs’ toxicity using silkworms’ qiufeng × baiyu strain. The concentrations of BN NSs were set at 2% and 8%, and the results showed that neither concentration had a lethal effect on silkworm growth (Figure 5, Table S8). All concentrations of BN NSs used had a beneficial impact on development in terms of length (Figure 5a,b,d,e) and weight (Figure 5c,f) compared to the control group as the days of administration proceeded. The study also found that no larvae died, even at the higher concentration of 8%, indicating that BN NSs are not toxic to silkworms in a wide range. 

In our previous study [18], inductively coupled plasma mass spectrometry (ICP-MS) was used to detect whether BN NSs were absorbed and accumulated in the silkworm body. The result showed no substantial accumulation of B elements in silkworm organs, despite continued intake of BN NSs, and that silkworms easily cleared the BN NSs. These findings suggest that BN NSs are not likely to degrade in the silkworm. Moreover, some researchers have suggested that BN NSs are thermally and chemically stable for a long time at high temperatures, making them impossible to degrade in the silkworm [32,33]. In summary, this and previous studies suggest that BN NSs are generally non-detrimental to the larval growth of the two silkworm strains within the studied dose range. 

### 3.3. The Influence of BN NSs on the Cocoons

**Figure 6:**
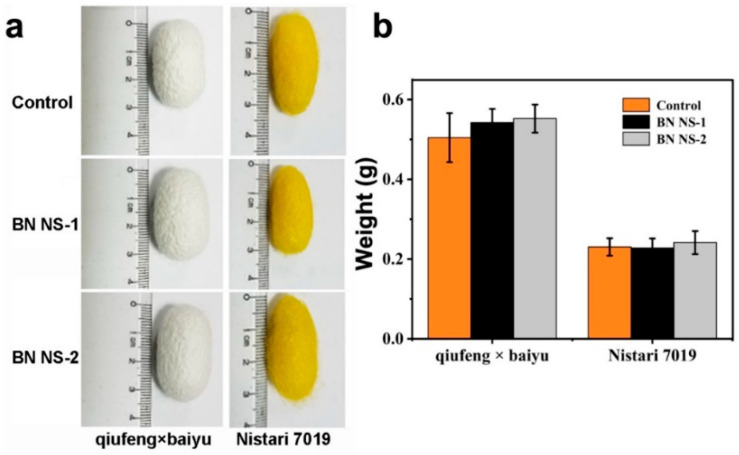
The external appearance of cocoons (**a**) and cocoon weight (**b**) from qiufeng × baiyu and Nistari 7019 silkworms after the intake of BN NSs-1 and BN NSs-2.

To assess the effect of the distinct sizes of BN NSs of the as-obtained cocoons, the appearance, size, and average weight were recorded. Figure 6 and Table S9 depict the size, physical characteristics, and average weight of cocoons from qiufeng × baiyu and Nistari 7019 at different concentrations of BN NSs (control (0% concentration), G1 (4% concentration each), and G2 (4% concentration)). A uniform cocoon appearance was observed in the groups of both strains (Figure 5a); however, the sizes and weights differed (Figure 5b). The average weight of qiufeng × baiyu cocoons from G1 (0.54 g) and G2 (0.55 g) was higher than that of the control (0.51 g) after being fed BN NSs (Figure 5b), indicating improved nutrient assimilation and transport. However, for Nistari 7019 cocoons, the control levels (0.23 g) and G2 (0.24 g) were higher than those of the G1 (0.22 g). It was observed that BN NSs had a higher effect on the growth rate of qiufeng × baiyu compared to Nistari 7019, demonstrating that the same treatment did not favorably affect the Nistari 7019 strain, as there was no significant increase in cocoon weight. It is possible that the Nistari 7019 strain may have a different nutritional requirement or may not respond well to the specific treatment used. Overall, the results suggest that the application of BN NSs positively affects the size and weight of cocoons; however, the effect may vary depending on the silkworm strain. BN NSs treatment may be a promising approach for improving the economic value of the qiufeng × baiyu strain, potentially leading to increased profitability for breeders. However, further research may be needed to better understand the mechanisms behind these effects and determine whether the treatment is effective for other strains.

The skewness Kurtosis test was used to evaluate the weight data of the cocoons obtained from qiufeng × baiyu and Nistari 7019 strains to determine whether they obeyed the normal distribution. Figure S6 showed that the weight data of the qiufeng × baiyu cocoon did not follow the normal distribution. To further investigate the differences in cocoon weight, the Kruskal–Wallis test was then used to determine whether there were statistically significant differences between the control group, G1, and G2 groups for both the qiufeng × baiyu and the Nistari 7019 strains. As shown in Tables S10 and S11, there were significant differences in cocoon weight among the three groups of qiufeng × baiyu but no significant differences in cocoon weight among the three groups of Nistari 7019. However, a larger dataset will be needed to validate the results. Apart from the size difference of the BN NSs and their influence on the interactions with biological systems, the differences between the strains of silkworms may have been another factor resulting in the differences in the cocoon weights. To begin, Nistari 7019 is a yellow blood silkworm, whereas qiufeng × baiyu is a white blood silkworm, and their blood composition differs significantly, including as regards proteins, amino acids, minerals, vitamins, carotenoids, lutein, and other pigments. The pigments of colored cocoons, such as the yellow cocoon, are derived from carotenoids [34,35]. Meanwhile, the inactivation of transposon-associated carotenoid-binding protein yields white cocoons and colorless hemolymph [36]. Second, the physiological and biochemical mechanism of silkworms spitting out colored cocoon silk has been primarily reported in the process of carotenoids and lutein transport from hemolymph to the middle silk gland, which pigment-binding proteins may accomplish. The pigments determine the color of cocoon silk in cocoon sericin, such as carotenoids and lutein, and genes [37]. Third, some studies have shown that the cocoon silk from Nistari 7019 has more internal pores than that from qiufeng × baiyu, and the internal fibril surface structure is also tighter; the characteristic peaks of infrared absorption spectrum are similar, indicating that their molecular conformations are similar; X-ray diffraction curves show that there is no noticeable difference in their crystal structure [38]. In summary, it is hypothesized that these differences between qiufeng × baiyu and Nistari 7019 may have caused the variations in weight and size of the cocoon. 

### 3.4. Histopathological Evaluation of Silkworm Tissues

**Figure 7:**
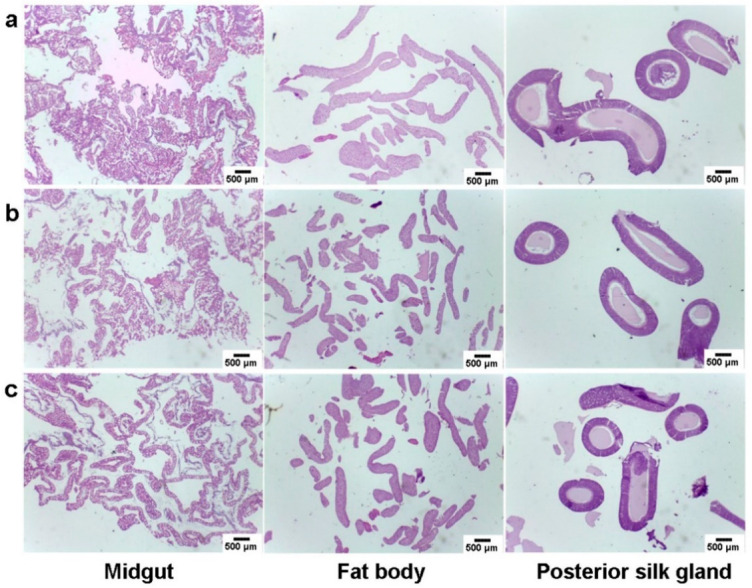
Histophysiological images of qiufeng × baiyu silkworm tissues: (**a**) control, (**b**) G1, and (**c**) G2 after the intake of BN NSs for 96 h.

**Figure 8:**
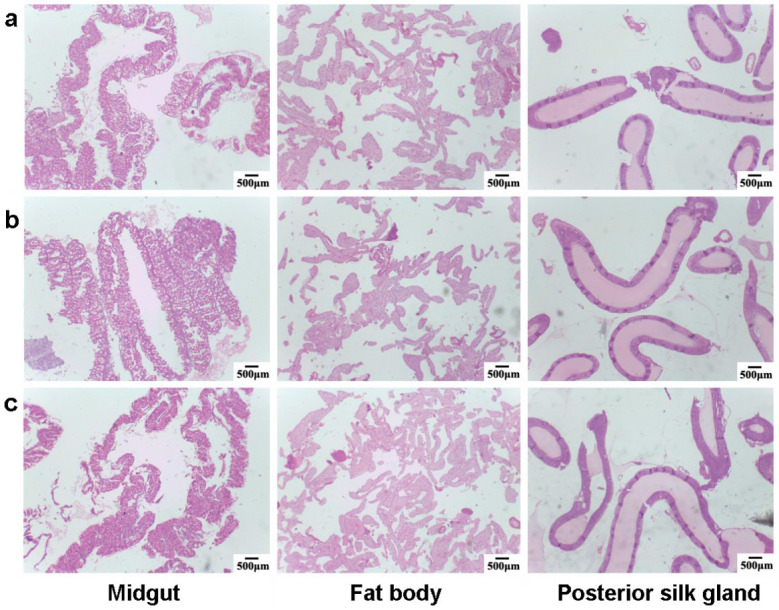
Histophysiological images of Nistari 7019 silkworm tissues: (**a**) control, (**b**) G1, and (**c**) G2 after the intake of BN NSs for 96 h.

In order to assess the effect of feeding BN NSs on the silkworm tissues, a histophysiological study was performed on the midgut, fat body, and posterior silk gland 96 h after the intake of BN NSs. The tissue microstructure for qiufeng × baiyu (Figure 7) and Nistari 7019 (Figure 8) was normal, and the size and cell morphology were consistent across all groups. In the midgut tissues, fully developed epithelial cells with columnar, cupped, and regenerative cells were easily distinguished. The structure of the fat body was clear and discernible, with a regular spindle shape, and was primarily distributed in the periphery of the digestive tract and other organs. Both the control and treatment groups of silk glands exhibited full gland lumen, thin walls, and standard architecture with preserved morphology. Therefore, it can be concluded that the consumption of BN NSs did not cause any harm to the silkworm tissues, and the histophysiological study demonstrated that the tissues remained normal after 96 h of intake. 

## 4. Conclusions

Our study presented an efficient method for assessing the effect of size and concentration of BN NSs using two silkworm strains (qiufeng × baiyu and Nistari 7019) as model organisms. The two distinct sizes of BN NSs (BN NSs-1, thickness of 41.5 nm and diameter of 270.7 nm; BN NSs-2, thickness of 48.2 nm, and diameter of 562.2 nm) did not show any significant adverse effect on the growth status of the larvae, cocoon appearance, weight, or cell morphology of silkworm tissues. However, while both BN NSs employed promoted the growth of qiufeng × baiyu, BN NSs-1 did not favorably impact the weight of both the larvae and cocoons of the Nistari 7019 strain. It is worth noting that the effects of nanomaterials on biological systems are complex and can influence a variety of factors. Therefore, further research is needed to fully understand the mechanisms underlying the effects of BN NSs-1 and BN NSs-2 on silkworms’ weight. In the present study, though there were no death records, at 8% and 96 h after feeding, the average weight of qiufeng × baiyu silkworm of both BN NS-1 and BN NSs-2 was lower than that of 2 and 4%, meaning an increase in concentration could lead to a further decrease in the body weight. Moreover, in our previous report [21], we found that though there were no records of death nor any adverse effect on the growth and tissues of jingsong × haoyue silkworm at a 4% concentration of BN NSs with an average diameter of 1.8 µm, significant changes in the expression of genes in the midgut involved in some specific functions were observed, indicating a potential risk of BN NSs causing dysfunction. Studying more diverse sizes and shapes is crucial to obtain a general regularity of size and shape-dependent effect. Therefore, further research on this topic will contribute to a better understanding of the relationship between the physiochemical properties of BN NSs and their impact, thereby informing the development of safe and effective nanomaterials for a variety of applications, as well as the environmental effects of their residues on nontarget organisms.

## Data Availability

Not applicable.

## Supplementary Materials

The following are available online at https://www.mdpi.com/article/10.3390/nano13091502/s1, Figure S1: Size distribution of BN NSs-1 (a) and BN NSs-2 (b). DLS data (Figure S1) show that the diameter of BN NSs-1 (Figure S1a) ranges from 164.2 nm to 458.7 nm, with an average diameter of 270.7 nm, and BN NSs-2 ranges from 458.7 nm to 712.4 nm, with an average diameter of 562.2 nm. BN NSs-1 and BN NSs-2 have PDI of 0.295 and 0.863, respectively, Table S1: The average weight and length of silkworm larvae (qiufeng × baiyu), Figure S2: One sample Kolmogorov–Smirnov test used to assess the data of silkworm larvae (qiufeng × baiyu) weight, Table S2: Kruskal–Wallis test used to assess the data of silkworm larvae (qiufeng × baiyu) weight from different groups (g) by changing time, Table S3: The average weight and length of silkworm larvae (Nistari 7019), Figure S3: One sample Kolmogorov–Smirnov test used to assess the data of silkworm larvae (Nistari 7019) weight, Table S4: Kruskal–Wallis test used to assess the data of silkworm larvae (Nistari 7019) weight from different groups (g) by changing time, Figure S4: One sample Kolmogorov–Smirnov test used to assess the data of silkworm larvae (qiufeng × baiyu) length, Table S5: Kruskal–Wallis Test for the data of silkworm larvae (qiufeng × baiyu) length from different groups (cm) by changing time, Figure S5: One sample Kolmogorov–Smirnov Test used to assess the data of silkworm larvae (Nistari 7019) length, Table S6: Kruskal–Wallis Test used to assess the data of silkworm larvae (Nistari 7019) length from different groups (cm) by changing time, Table S7: The raw data of weight of mulberry leaves eaten by silkworms (qiufeng × baiyu) (g), Table S8: The average weight and length of qiufeng × baiyu at different concentrations, Table S9: The weight of cocoon from qiufeng × baiyu and Nistari 7019 from different groups (g), Figure S6: One sample Kolmogorov–Smirnov test used to assess the cocoon weight data from silkworm larvae (qiufeng × baiyu, Nistari 7019), Table S10: Kruskal–Wallis Test used to assess the data of cocoon (qiufeng × baiyu) weight from different groups (g), Table S11: Kruskal–Wallis Test used to assess the data of cocoon (Nistari 7019) weight from different groups (g).
