# Supplementary material for: The Influence of the Size of BN NSs on Silkworm Development and Tissue Microstructure
Source: Nanomaterials (Basel). 2023 Apr 27;13(9):1502. doi: 10.3390/nano13091502 (PMC10180518; doi:10.3390/nano13091502)
Supplement: Supplementary file 1 [file nanomaterials-13-01502-s001.zip › nanomaterials-2354032-supplementary.pdf]

Supplementary Materials

The Influence of the Size of BN NSs on Silkworm Development and Tissue Microstructure

Vivian Andoh [1,2,†], Haiyan Liu [3,†], Liang Chen [2], Lin Ma [4,*] and Keping Chen [1,2,*]

[1] School of Food and Biological Engineering, Jiangsu University, Zhenjiang 212013, China;
vandoh@ujs.edu.cn
[2] School of Life Sciences, Jiangsu University, Zhenjiang 212013, China;
oochen@ujs.edu.cn
[3] College of Tea and Food Science Technology, Jiangsu Vocational College of Agriculture and Forestry,
Jurong 212400, China; yanliuhai@126.com
[4] College of Biotechnology, Jiangsu University of Science and Technology, Zhenjiang 212000, China
* Correspondence: ma_lin_1988@126.com (L.M.); kpchen@ujs.edu.cn (K.C.)
† These authors contributed equally to this work.

Results and Discussions

Size distribution of BN NSs

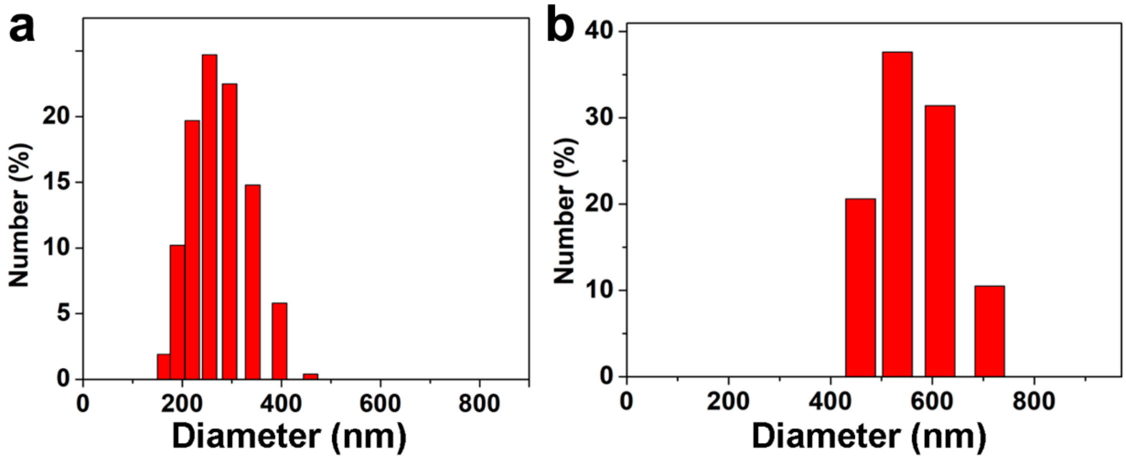

Figure S1. Size distribution of BN NSs-1 a) and BN NSs-2 b).

DLS data (Figure S1) show that the diameter of BN NSs-1 (Figure S1a) ranges from 164.2 nm to 458.7 nm, with an average diameter of 270.7 nm, and BN NSs-2 ranges from 458.7 nm to 712.4 nm, with an average diameter of 562.2 nm. BN NSs-1 and BN NSs-2 have PDI of 0.295 and 0.863, respectively.

Statistical Analysis

Table S1. The average weight and length of silkworm larvae (qiufeng × baiyu).

Sample	Average Weight (g)	Average Length (cm)
Control (0 h)	0.576	4.1
G1 (0 h)	0.553	4.0
G2 (0 h)	0.594	4.1
Control (24 h)	1.040	5.0
G1 (24 h)	0.901	4.7
G2 (24 h)	1.094	5.1
Control (48 h)	1.613	5.7
G1 (48 h)	1.610	5.6

G2 (48 h)	1.665	5.8
Control (72 h)	2.381	6.4
G1 (72 h)	2.511	6.5
G2 (72 h)	2.503	6.5
Control (96 h)	2.932	6.8
G1 (96 h)	3.082	6.9
G2 (96 h)	3.078	6.9

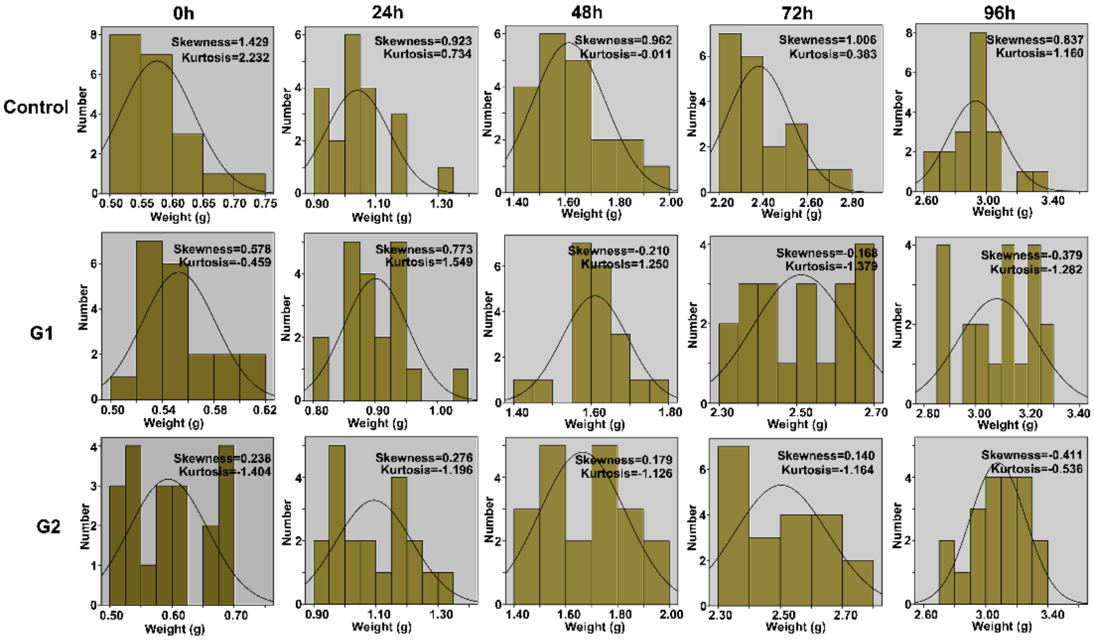

Figure S2. One sample Kolmogorov–Smirnov test used to assess the data of silkworm larvae (qiufeng × baiyu) weight.

Table S2. Kruskal–Wallis test used to assess the data of silkworm larvae (qiufeng × baiyu) weight from different groups (g) by changing time.

		Number	Average Grade	X^2	df	Progressive Significance
**0h**	**Control**	20	31.25	3.568	2	0.168
	**G1**	20	24.95			
	**G2**	20	35.30			

	**Total**	60				
**24h**	**Control**	20	36.28	31.191	2	0
	**G1**	20	13.03			
	**G2**	20	42.20			
	**Total**	60				
**48h**	**Control**	20	27.35	1.555	2	0.46
	**G1**	20	29.98			
	**G2**	20	34.18			
	**Total**	60				
**72h**	**Control**	20	20.65	9.639	2	0.008
	**G1**	20	36.28			
	**G2**	20	34.58			
	**Total**	60				
**96h**	**Control**	20	20.63	9.615	2	0.008
	**G1**	20	35.85			
	**G2**	20	35.03			
	**Total**	60				

Note: When the degree of freedom (df) is 2, and the confidence level (α) is 0.05, the critical value of X^2 is 5.991 by referring to the X^2 distribution critical value table. X^2 value is less than 5.991, and the progressive significance value is larger than 0.05, indicating that the conclusion is to accept the null hypothesis, and there is no significant difference among the three groups. X^2 value is larger than 5.991, and the progressive significance value is less than 0.05, indicating that the conclusion is to reject the null hypothesis, and there is a significant difference among the three groups.

Table S3. The average weight and length of silkworm larvae (Nistari 7019).

Sample	Average Weight (g)	Average Length (cm)
Control (0 h)	0.407	3.8
G1 (0 h)	0.436	3.8
G2 (0 h)	0.419	3.9
Control (24 h)	0.543	4.2
G1 (24 h)	0.564	4.4
G2 (24 h)	0.557	4.4

Control (48 h)	0.786	5.0
G1 (48 h)	0.798	5.0
G2 (48 h)	0.841	5.0
Control (72 h)	1.056	5.4
G1 (72 h)	0.997	5.3
G2 (72 h)	1.067	5.4
Control (96 h)	1.242	5.7
G1 (96 h)	1.192	5.7
G2 (96 h)	1.283	5.8

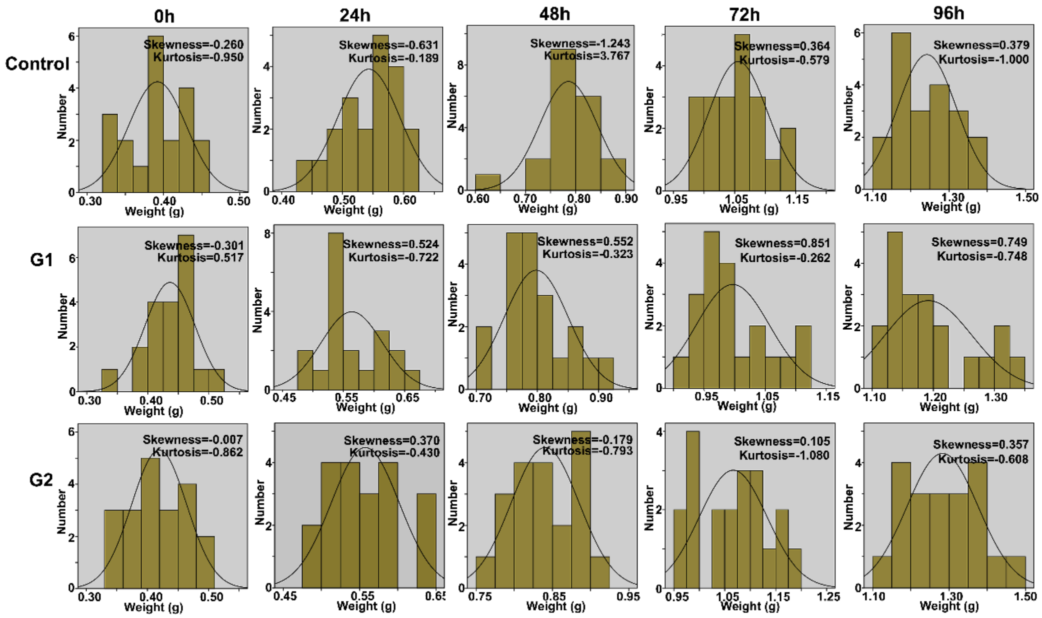

Figure S3. One sample Kolmogorov–Smirnov test used to assess the data of silkworm larvae (Nistari 7019) weight.

Table S4. Kruskal–Wallis test used to assess the data of silkworm larvae (Nistari 7019) weight from different groups (g) by changing time.

		Number	Average Grade	X^2	df	Progressive Significance
**0h**	**Control**	20	24.60	5.032	2	0.081
	**G1**	20	36.95			
	**G2**	20	29.95			
	**Total**	60	-			
**24h**	**Control**	20	27.93	0.739	2	0.691
	**G1**	20	32.60			
	**G2**	20	30.98			
	**Total**	60	-			
**48h**	**Control**	20	24.33	10.434	2	0.005
	**G1**	20	26.45			
	**G2**	20	40.73			
	**Total**	60	-			
**72h**	**Control**	20	35.43	14.07	2	0.001
	**G1**	20	18.60			
	**G2**	20	37.48			
	**Total**	60	-			
**96h**	**Control**	20	32.20	11.568	2	0.003
	**G1**	20	20.38			
	**G2**	20	38.93			
	**Total**	60	-			

Note: When the degree of freedom (df) is 2, and the confidence level (α) is 0.05, the critical value of X^2 is 5.991 by referring to the X^2 distribution critical value table. X^2 value is less than 5.991, and the progressive significance value is larger than 0.05, indicating that the conclusion is to accept the null hypothesis, and there is no significant difference among the three groups. X^2 value is larger than 5.991, and the progressive significance value is less than 0.05, indicating that the conclusion is to reject the null hypothesis, and there is a significant difference among the three groups.

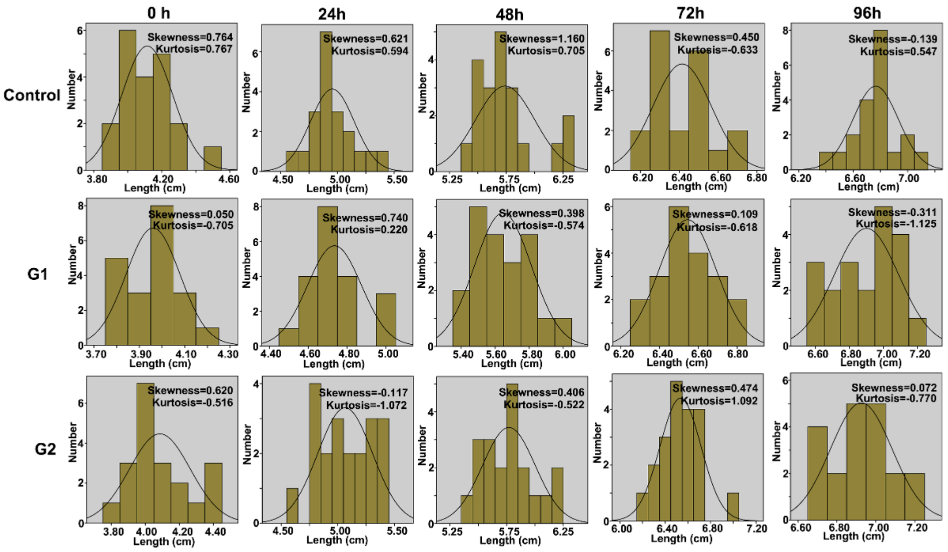

Figure S4. One sample Kolmogorov–Smirnov test used to assess the data of silkworm larvae (qiufeng × baiyu) length.

Table S5. Kruskal–Wallis Test for the data of silkworm larvae (qiufeng × baiyu) length from different groups (cm) by changing time

		Number	Average Grade	X^2	df	Progressive Significance
**0h**	**Control**	20	35.00	4.355	2	0.113
	**G1**	20	24.25			
	**G2**	20	32.25			
	**Total**	60				
**24h**	**Control**	20	34.43	21.544	2	0
	**G1**	20	16.30			
	**G2**	20	40.78			
	**Total**	60				
**48h**	**Control**	20	31.03	2.923	2	0.232
	**G1**	20	25.60			
	**G2**	20	34.88			
	**Total**	60				
**72h**	**Control**	20	22.00	7.479	2	0.024
	**G1**	20	35.58			
	**G2**	20	33.93			
	**Total**	60				
**96h**	**Control**	20	21.63	8.102	2	0.017
	**G1**	20	33.93			
	**G2**	20	35.95			
	**Total**	60				

Note: When the degree of freedom (df) is 2, and the confidence level (α) is 0.05, the critical value of X^2 is 5.991 by referring to the X^2 distribution critical value table. X^2 value is less than 5.991, and the progressive significance value is larger than 0.05, indicating that the conclusion is to accept the null hypothesis, and there is no significant difference among the three groups. X^2 value is larger than 5.991, and the progressive significance value is less than 0.05, indicating that the conclusion is to reject the null hypothesis, and there is a significant difference among the three groups.

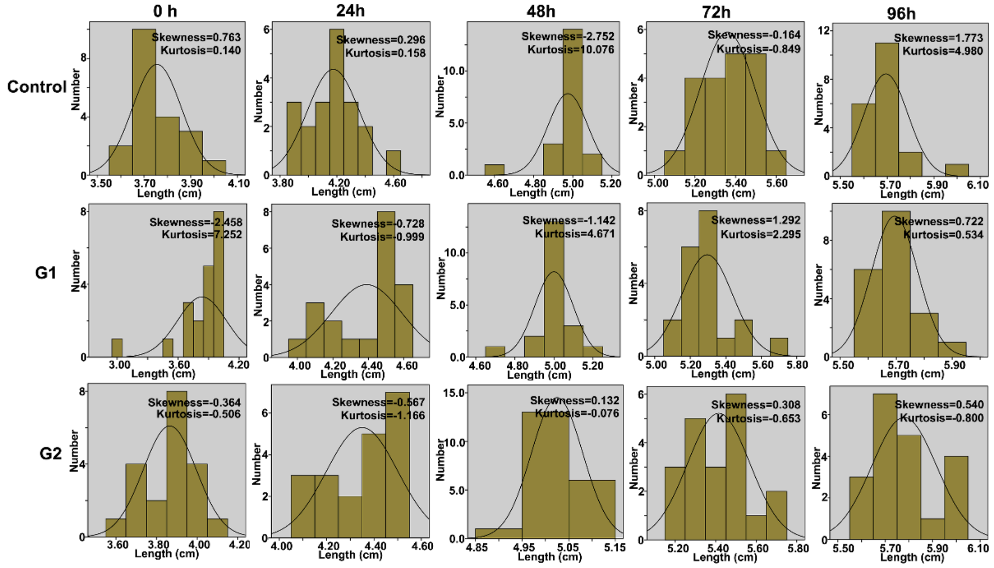

Figure S5. One sample Kolmogorov–Smirnov Test used to assess the data of silkworm larvae (Nistari 7019) length.

Table S6. Kruskal–Wallis Test used to assess the data of silkworm larvae (Nistari 7019) length from different groups (cm) by changing time.

		**Number**	**Average Grade**	X^2	**df**	**Progressive Significance**
**0h**	**Control**	20	25.50	2.682	2	0.262
	**G1**	20	33.70			
	**G2**	20	32.30			
	**Total**	60				
**24h**	**Control**	20	19.73	12.423	2	0.002
	**G1**	20	38.10			
	**G2**	20	33.68			

	**Total**	60				
**48h**	**Control**	20	26.23	3.631	2	0.163
	**G1**	20	30.28			
	**G2**	20	35.00			
	**Total**	60				
**72h**	**Control**	20	31.68	6.81	2	0.033
	**G1**	20	22.95			
	**G2**	20	36.88			
	**Total**	60				
**96h**	**Control**	20	26.38	6.284	2	0.043
	**G1**	20	27.15			
	**G2**	20	37.98			
	**Total**	60	-			

Note: When the degree of freedom (df) is 2, and the confidence level (α) is 0.05, the critical value of X^2 is 5.991 by referring to the X^2 distribution critical value table. X^2 value is less than 5.991, and the progressive significance value is larger than 0.05, indicating that the conclusion is to accept the null hypothesis, and there is no significant difference among the three groups. X^2 value is larger than 5.991, and the progressive significance value is less than 0.05, indicating that the conclusion is to reject the null hypothesis, and there is a significant difference among the three groups.

The Food Intake Speed of Silkworms

To study the food intake rate of silkworms, the daily weight of mulberry leaves consumed by silkworms was measured using qiufeng × baiyu as a model and two concentrations (2% and 8%) of BN NSs-1 and BN NSs-2. The data in **Table S7** indicate a subtle distinction between the groups of silkworms in terms of their food intake rate. Future research should focus on each silkworm's food consumption rate within each group.

Table S7. The raw data of weight of mulberry leaves eaten by silkworms (qiufeng × baiyu) (g).

	control	BN NSs-1		BN NSs-2	
		2%	8%	2%	8%
**1st**	47.47	47.96	46.45	51.44	44.83
**2nd**	42.02	46.1	49.61	48.48	47.24
**3rd**	43.51	43.32	43.77	44.73	43.75
**4th**	46.89	49.96	45.29	46.28	43.94

The Concentration and Time Effect of BN NSs Toxicity

Table S8. The average weight and length of qiufeng × baiyu at different concentrations.

Sample	BN NS-1		BN NSs-2	
	Average Weight (g)	Average Length (cm)	Average Weight (g)	Average Length (cm)
Control (24 h)	0.825	4.3	0.825	4.3
2% (24 h)	0.954	4.3	1.028	4.5
8% (24 h)	0.944	4.4	1.011	4.6
Control (48 h)	1.263	4.8	1.263	4.8
2% (48 h)	1.347	5.0	1.408	5.1
8% (48 h)	1.478	5.2	1.390	5.1
Control (72 h)	1.629	5.3	1.629	5.3
2% (72 h)	1.679	5.2	1.716	5.4
8% (72 h)	1.913	5.5	1.949	5.6
Control (96 h)	2.020	5.7	2.020	5.7
2% (96 h)	2.272	6.1	2.114	5.7
8% (96 h)	2.322	6.0	2.418	5.8

Table S9. The weight of cocoon from qiufeng × baiyu and Nistari 7019 from different groups (g).

**Sample**	**qiufeng × baiyu**			**Nistari 7019**		
	**Control**	**G1**	**G2**	**Control**	**G1**	**G2**
1	0.507	0.562	0.524	0.196	0.211	0.254
2	0.473	0.549	0.593	0.214	0.216	0.311
3	0.535	0.499	0.536	0.224	0.259	0.241
4	0.567	0.529	0.578	0.213	0.274	0.249
5	0.542	0.547	0.548	0.232	0.224	0.223
6	0.566	0.507	0.523	0.225	0.203	0.236
7	0.384	0.567	0.524	0.282	0.229	0.254
8	0.429	0.566	0.545	0.253	0.208	0.234
9	0.484	0.531	0.529	0.248	0.223	0.259
10	0.466	0.569	0.525	0.237	0.204	0.203
11	0.558	0.57	0.603	0.249	0.227	0.279
12	0.449	0.538	0.54	0.252	0.208	0.234
13	0.526	0.574	0.525	0.237	0.205	0.258
14	0.353	0.571	0.563	0.203	0.213	0.214
15	0.532	0.529	0.493	0.232	0.218	0.268
16	0.537	0.431	0.62	0.241	0.258	0.265
17	0.543	0.577	0.582	0.204	0.254	0.24
18	0.532	0.56	0.578	0.248	0.271	0.201
19	0.54	0.537	0.515	0.204	0.247	0.202
20	0.567	0.532	0.601	0.212	0.202	0.202
**Average**	**0.505**	**0.542**	**0.552**	**0.23**	**0.228**	**0.241**
**Standard** deviation	**0.061**	**0.034**	**0.035**	**0.022**	**0.024**	**0.029**

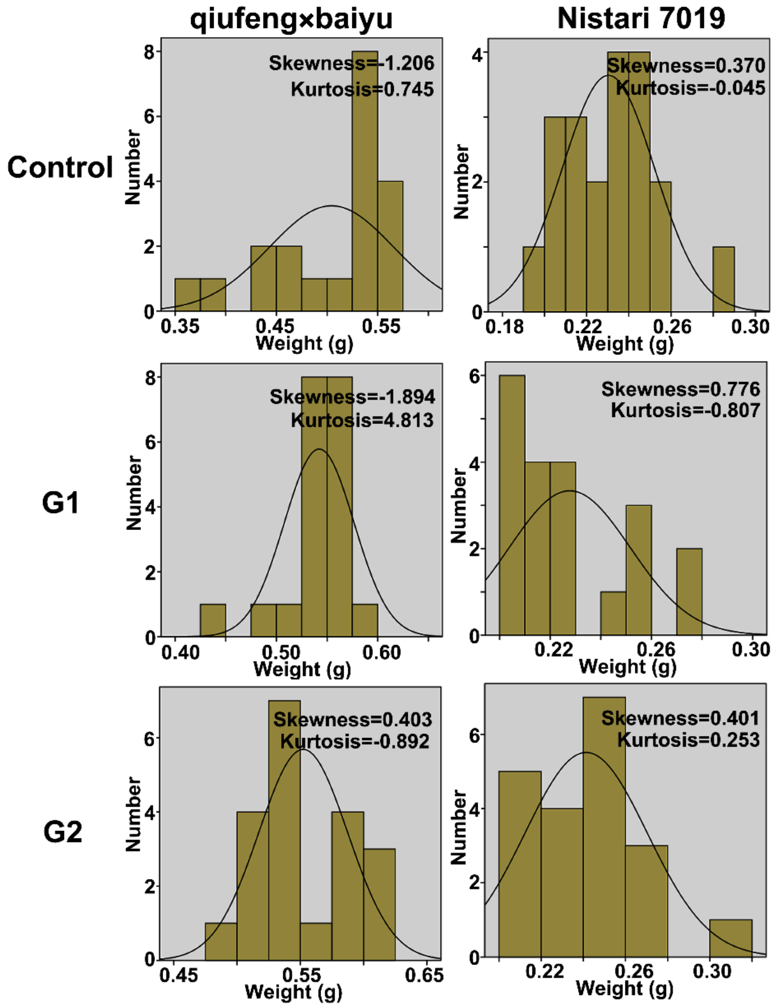

Figure S6: One sample Kolmogorov–Smirnov test used to assess the cocoon weight data from silkworm larvae (qiufeng × baiyu, Nistari 7019).

Table S10: Kruskal–Wallis Test used to assess the data of cocoon (qiufeng × baiyu) weight from different groups (g)

		**Number**	**Average grade**	X^2	**df**	**Progressive significance**
**Weight**	**Control**	20	22.45	6.404	2	0.041
	**G1**	20	34.08			
	**G2**	20	34.98			
	**Total**	60	-			

Note: When the degree of freedom (df) is 2, and the confidence level (α) is 0.05, the critical value of X^2 is 5.991 by referring to the X^2 distribution critical value table. X^2 value is less than 5.991, and the progressive significance value is larger than 0.05,

indicating that the conclusion is to accept the null hypothesis, and there is no significant difference among the three groups. X^2 value is larger than 5.991, and the progressive significance value is less than 0.05, indicating that the conclusion is to reject the null hypothesis, and there is a significant difference among the three groups.

Table S11: Kruskal–Wallis Test used to assess the data of cocoon (Nistari 7019) weight from different groups (g).

		Number	Average grade	X^2	df	Progressive significance
Weight	Control	20	29.03	2.275	2	0.321
	G1	20	27.28			
	G2	20	35.20			
	Total	60	-			

Note: When the degree of freedom (df) is 2, and the confidence level (α) is 0.05, the critical value of X^2 is 5.991 by referring to the X^2 distribution critical value table. X^2 value is less than 5.991, and the progressive significance value is larger than 0.05, indicating that the conclusion is to accept the null hypothesis, and there is no significant difference among the three groups. X^2 value is larger than 5.991, and the progressive significance value is less than 0.05, indicating that the conclusion is to reject the null hypothesis, and there is a significant difference among the three groups.
